# Supplementary material for: Interaction Analysis between the Arabidopsis Transcription Repressor VAL1 and Transcription Coregulators SIN3-LIKEs (SNLs)
Source: Int J Mol Sci. 2022 Jun 23;23(13):6987. doi: 10.3390/ijms23136987 (PMC9266683; doi:10.3390/ijms23136987)
Supplement: Supplementary file 1 [file ijms-23-06987-s001.zip › ijms-23-06987-s001/ijms-1740261-supplementary.pdf]

Supplemental Information for

Interaction analysis between the *Arabidopsis* transcription repressor VAL1 and transcription coregulators SIN3-LIKEs (SNLs)

Chuanyou Chen[1+], Xia Gong[1+], Yan Li[1], Haitao Li[1], Haitao Zhang[1], Li Liu[1], Dacheng Liang[2], Wenya Yuan[1*]

[1]State Key Laboratory of Biocatalysis and Enzyme Engineering, School of Life Sciences, Hubei University, Wuhan 430062, China

[2]Hubei Collaborative Innovation Center for Grain Industry, School of Agriculture, Yangtze University, Jingzhou 434023, China

[+]These authors contributed equally to this work.

[*]Coresponding authors: Wenya Yuan

Email: wyyuan@hubu.edu.cn

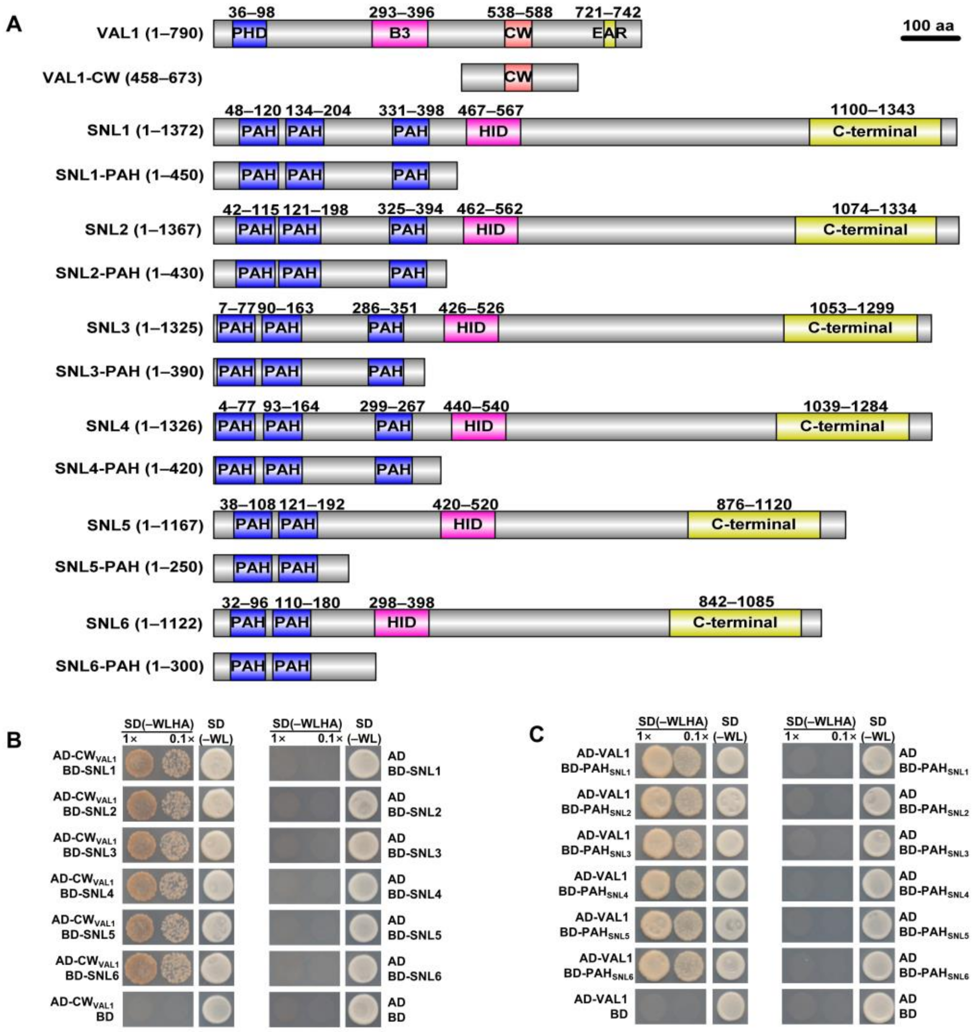

Figure S1. VAL1 interacts with SNLs through the CW domain of VAL1 and the PAH domains of SNLs in yeast cells. (A) Schematic presentation of VAL1 and SNLs (SNL1–SNL6), and the CW domain of VAL1 and the PAH domains of SNLs. Bar = 100 aa. (B) The CW domain of VAL1 (aa 458–673) interacts with intact SNLs in yeast cells. The CW domain of *VAL1* and intact *SNLs* were fused to the GAL4 activating domain (AD) and binding domain (BD), respectively. The transformed yeast cells were spotted onto a stringent selection medium lacking Trp, Leu, His, and Ade (–WLHA) or a non-selective medium lacking Trp and Leu (–WL; control). (C) Intact VAL1 interacts with the PAH domains of SNLs in yeast cells. Intact *VAL1* and the PAH domains of *SNLs* were fused to the GAL4 activating domain (AD) and binding domain (BD), respectively. The transformed yeast cells were spotted onto a stringent selection medium lacking Trp, Leu, His, and Ade (–WLHA) or a non-selective medium lacking Trp and Leu (–WL; control).

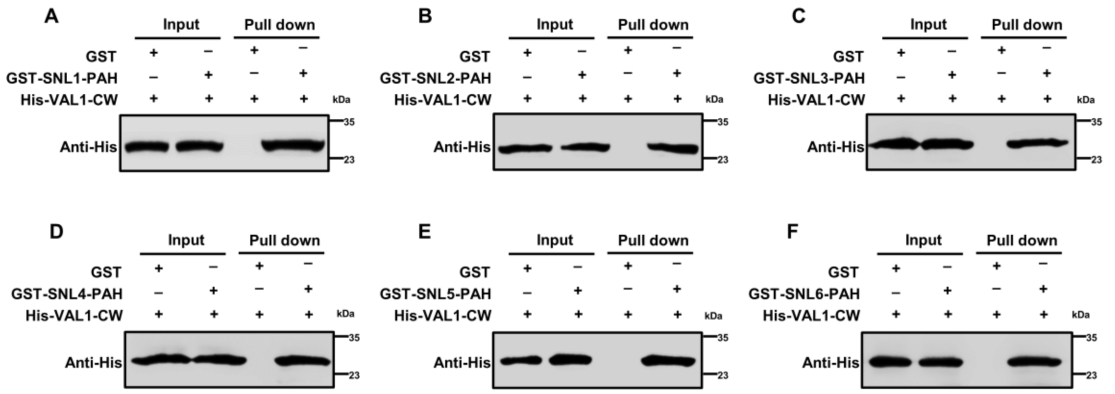

Figure S2. In vitro pull-down assay validated that the CW domain of VAL1 interacts with the PAH domains of SNLs. (A–F) Induced His-VAL1-CW (aa 458–673) was incubated with the GST-SNLs-PAH (SNL1-PAH, aa 1–450; SNL2-PAH, aa 1–430; SNL3-PAH, aa 1–390; SNL4-PAH, aa 1–430; SNL5-PAH, aa 1–250; SNL6-PAH, aa 1–300) or GST protein. Protein samples were immunoprecipitated with anti-GST antibodies and immunoblotted with anti-His antibodies. The symbols "−"and "+" represent the absence and presence of corresponding proteins, respectively.

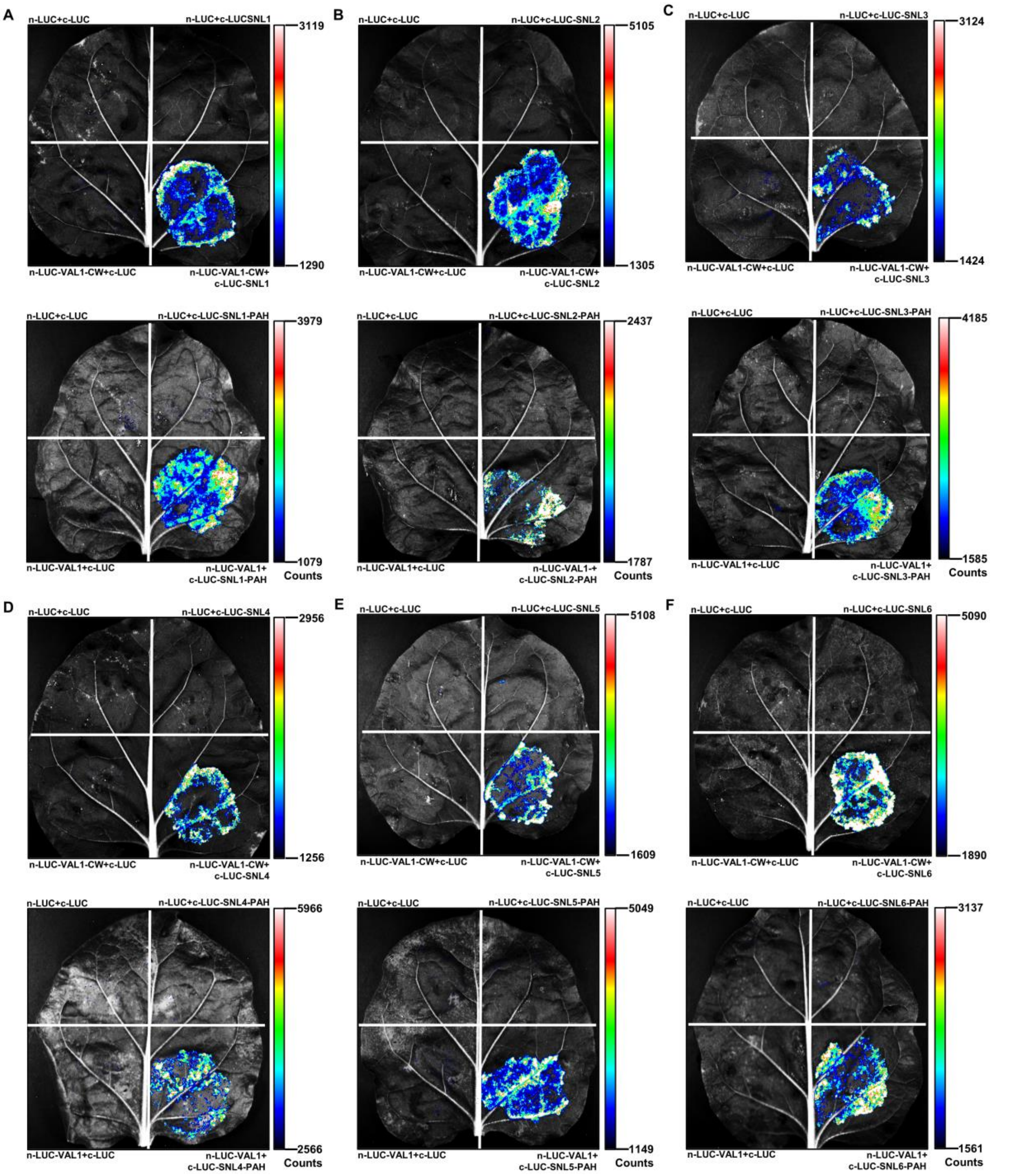

Figure S3. LCI assays verified that VAL1 interacts with SNLs through the CW domain of VAL1 and the PAH domains of SNLs in tobacco. (A–F) The CW domain of VAL1 (aa 458–673) and the PAH domains of SNL1 (aa 1–450), SNL2 (aa 1–430), SNL3 (aa 1–390), SNL4 (aa 1–420), SNL5 (aa 1–250) and SNL6 (aa 1–300) interact with each other intact proteins in the leaf epidermal cells of *N. benthamiana*. The intact and CW domain of *VAL1* were fused to the *n-LUC* fragment, and the intact and PAH domains of *SNLs* (*SNL1–SNL6*) were fused to the *c-LUC* fragment.

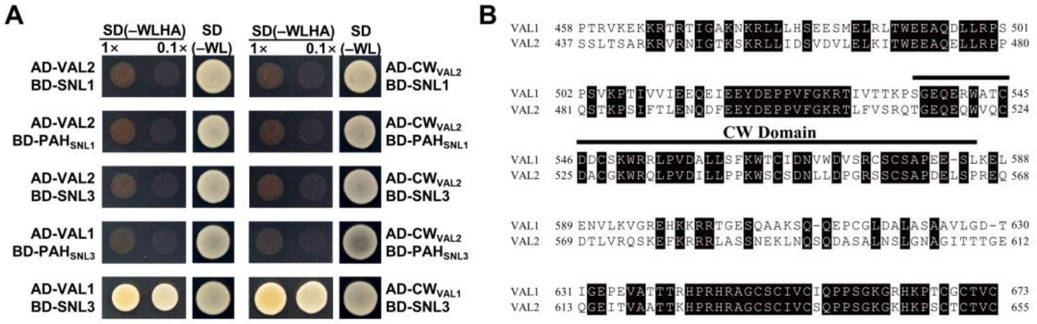

Figure S4. No interaction between VAL2 and SNL1/3 in yeast cells and A sequence alignment of VAL1-CW and VAL2-CW. (A) Intact VAL2 and the CW domain of VAL2 (aa 437–655) can not interact with intact SNL1/3 or the PAH domains of SNL1/3 in yeast cells. The full-length or regions of VAL1 and SNL1/3 were fused to the GAL4 activating domain (AD) and binding domain (BD), respectively. The transformed yeast cells were spotted onto a stringent selection medium lacking Trp, Leu, His, and Ade (−WLHA) or a non-selective medium lacking Trp and Leu (−WL; control). The pairwise combinations of VAL1/SNL3 and VAL1-CW/SNL3 were used as positive controls. (B) Amino acid sequence alignment of VAL1-CW (aa 458–673) and VAL2-CW (aa 437–655). The dark line shows the CW domains of VAL1 and VAL2.
